# Ranolazine-functionalized CuO NPs: efficient homogeneous and heterogeneous catalysts for reduction of 4-nitrophenol

**DOI:** 10.3906/kim-1909-22

**Published:** 2020-02-11

**Authors:** Gul Naz LAGHARI BALOCH, Sarfaraz Ahmed MAHESAR, Sirajuddin ., Jan NISAR, Syed Tufail Hussain SHERAZI

**Affiliations:** 1 National Centre of Excellence in Analytical Chemistry, University of Sindh, Jamshoro Pakistan; 2 International Center for Chemical and Biological Sciences, University of Karachi, Karachi Pakistan; 3 National Centre of Excellence in Physical Chemistry, University of Peshawar, Peshawar Pakistan

**Keywords:** Copper oxide nanoparticles, ranolazine, catalysis, reduction, 4-nitrophenol

## Abstract

In the present study copper oxide nanoparticles (CuO NPs) were synthesized using a hydrothermal method with ranolazine as a shape-directing agent. Ranolazine-functionalized CuO NPs were characterized by various analytical techniques such as scanning electron microscopy (SEM), Fourier transform infrared (FTIR) spectroscopy and X-ray diffraction (XRD). The SEM pattern confirmed the morphology of ranolazine-functionalized CuO NPs with well-defined rice-like structures. FTIR spectroscopy confirmed the interaction between CuO NPs and ranolazine. The XRD analysis indicated that the structure of ranolazine-functionalized CuO NPs was monoclinic crystalline and the size ranged between 9 and 18 nm with an average particle size of 12 nm. The smaller size range of CuO NPs gave a large surface area that enhanced the efficiency of these catalysts employed for the reduction of 4-nitrophenol (4-NP) to 4-aminophenol (4-AP) in the H _2_ O system. In homogeneous catalysis, results showed that 50 μL of CuO NPs was required in the presence of NaBH4 for 99% reduction of 4-NP in 240 s. On the other hand, for heterogeneous catalysis, 0.5 mg of CuO NPs was used in the presence of NaBH4 for 99% catalytic reduction of 4-NP to 4-AP in 320 s. The rate of reaction for homogeneous catalysis and heterogeneous catalysis was determined from the plots of In(Ct /C0) of 4-NP versus time (s), which showed good linearity with values of 1.3 × 10 ^-2^ and 8.8 × 10 ^-3^ s ^-1^ . respectively. The high-quality catalytic efficiency, good reusability, nontoxic nature, and low cost are favorable properties of the synthesized CuO NPs for use as efficient catalysts for reduction of 4-AP to 4-NP in both homogeneous and heterogeneous media.

## 1. Introduction

Since the industrial revolution, environmental pollution has emerged as a serious issue for communities and researchers [1–3]. Many researchers are recommending green approaches to convert toxic compounds produced during industrial processing and spread in the environment to safe or less toxic compounds [4]. Various methods have been developed for the reduction of nitroaromatic compounds. 4-Nitrophenol (4-NP) is a common contaminant in industrial and agricultural wastewaters. It is an industrial impurity exhibiting significant solubility and stability in the water [5,6]. The US Environmental Protection Agency listed 4-NP as a notable contaminate that can be accumulated in different food chains and may have hazardous impacts on human health [7]. 4-NP is widely used as a precursor for manufacturing 4-AP, which is used for developing photographic originators, as a corrosion inhibitor, for the amalgamation of special industrial colorants, and for ventilation, as well as the main intermediates of analgesic and antipyretic medications. Several procedures are present in the literature for the preparation of 4-aminophenol (4-AP), such as hydrogenation of nitrobenzene, catalytic amination of hydroquinone, and electrochemical reduction of nitrophenols [8]. Various methods for the removal of 4-NP include catalytic adsorption [9], microwave-assisted oxidation [10], electrocoagulation [11], microbial degradation, photocatalytic degradation, and electrochemical methods [12]. Catalytic hydrogenation of 4-NP involves toxic solvents such as ethanol, high temperatures, and high hydrogen pressure. Therefore, a greener, more stable, economic, and efficient catalytic route is required for the reduction of 4-NP to 4-AP.

Metal nanoparticles have gained much importance in the field of catalysis due to their high efficiency in catalytic processes. The smaller size of nanoparticles exhibits superior catalytic activities due to their large surface-to-volume ratio. The permanence of metal and metal oxide NPs has also been improved by using different capping agents to prevent aggregation and improve catalytic performance [13,14]. Yilmaz et al. followed a green solvothermal method for preparation of palladium NPs on graphene oxide and applied them for bifunctional catalysis [15]. Furthermore, the typical synthesis of Pd-modified magnetic Sm _2_ O _3_ –ZrO _2_ NPs was followed by deposition–precipitation methods and applied for bifunctional catalysis [16]. Copper oxide nanoparticles (CuO NPs) have several industrial as well as commercial applications. CuO NPs are used in different fields, such as ink additives, plastics, cosmetics, and lubricant products [17]. Furthermore, they also play an important role in gas sensors, catalysts, solar cells, lithium batteries, and semiconductors devices [18]. A variety of microorganisms such as bacteria, fungi, and actinomycetes and various plant extracts have been used for the green synthesis of nanoparticles. Nadaroglu et al. followed a green synthesis route for the preparation of Au NPs by egg yolk. These NPs were characterized using UV-Vis spectroscopy, XRD and SEM [19]. A previous study reported the preparation of TiO _2_ NPs by using Aspergillus niger and characterized these NPs by UV spectrum, XRD, and SEM techniques, but in some cases it is very difficult to control the growth of micrograms as well as typical the size of the nanoparticles [20]. Synthesis of CuO NPs was done by using different routes such as hydrothermal methods, sol-gel techniques, sonochemical protocols, and microemulsion procedures [21]. Another work applied a laser ablation route for the synthesis of synthesized CuO NPs in an aqueous medium [22]. Phiwdang et al. prepared CuO NPs by precipitation method and characterized these nanoparticles using XRD, SEM, and FTIR spectroscopy [23]. The hydrothermal procedure is a more convenient route for synthesizing CuO NPs. In the present study, a hydrothermal procedure was applied for the preparation of ranolazine-functionalized CuO NPs. Synthesized CuO NPs were used as homogeneous and heterogeneous catalysts for the reduction of 4-NP to 4-AP in an aqueous medium.

## 2. Experimental

### 2.1. Materials and methods

Copper acetate monohydrate (C _4_ H _6_ CuO _4_ .H _2_ O) and ammonia (33%) were purchased from Sigma-Aldrich. NaBH _4_ , 4-NP, and ranolazine (C _24_ H _33_ N _3_ O _4_ ) were purchased from Fluka Chemicals. All pure solvents and chemicals were used by employing Milli-Q water as the preparation system.

### 2.2. Instrumentation

The FTIR analysis employed a Nicolet 5700 Thermo FTIR spectrometer to confirm the surface interaction between CuO NPs and ranolazine after incorporating the dried sample in a solid state KBr disc. Scanning electron microscopy (model JSM-6380LV) was used to describe the morphological characterization for shape homogeneity of synthesized CuO NPs. X-ray diffraction (D-8 Bruker) was used to confirm the phase purity and crystalline nature of CuO NPs as well as the size of CuO NPs. UV-Vis spectroscopy (Lambda 356, PerkinElmer) was used for recording the reduction of 4-NP to 4-AP.

### 2.3. Synthesis protocol of CuO NPs

Synthesis of CuO NPs was carried out by preparing a mixture of 0.1 g of ranolazine and 1.7 g of copper acetate monohydrate in 95 mL of H _2_ O solution and it was stirred for at least 10 min. After that, 5 mL of ammonia was added dropwise into the solution and the reaction medium was enclosed with aluminum foil and moved to an oven at 95 ◦ C for about 4 h. After that, the mixture was filtered and washed three times with methanol and pure water. Finally, the black powder of CuO NPs was dried for 1 day and transferred to a capped test tube.

### 2.4. Procedure for catalytic reduction of 4-NP

In the homogeneous system, 4-NP (2 mL from 0.1 mM solution) was diluted in water (1.6 mL) in a quartz cell. Then 0.3 mL (10 mM) of NaBH4 was added and the color of the mixture turned deep yellow from pale yellow due to the conversion of nitrophenol to the nitrophenolate anion. This mixture was further treated by adding 20 μL (0.1 mg mL ^-1^ ) and 50 μL (0.25 mg mL ^-1^ ) of CuO NPs. The deep yellow solution changed to a colorless solution, which indicated the catalytic reduction of 4-NP. In the heterogeneous system, two pieces of preweighed glass slides were assembled with CuO NPs at doses of 0.2 and 0.5 mg mL ^-1^ and placed in the solution of 4-NP. Like the homogeneous protocol, dilution with water (1.6 mL) and addition of 0.3 mL (10 mM) of NaBH4 was performed in a quartz cuvette. The changes were observed by UV-Vis spectrometry.

### 2.5. Reusability of recoverable catalyst

Glass slides on which CuO NPs were deposited and used for the reduction of 4-NP were taken out of the cuvette cell, washed slowly with pure water, and dried for further use. Similarly, these particles were reused for up to 5 cycles and their catalytic efficiency was measured.

## 3. Results and discussion

### 3.1. Infrared spectroscopy

**Figure 1:**
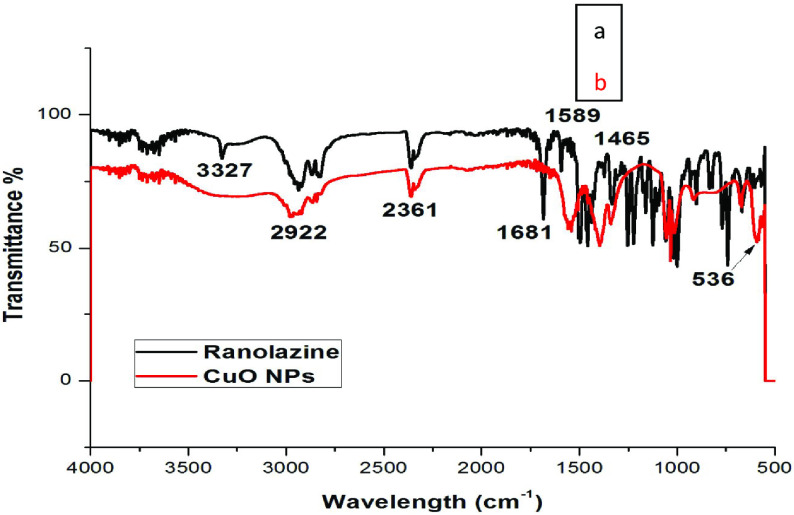
FTIR spectrum of (a) pure ranolazine and (b) ranolazine functionalized CuO NPs.

FTIR study confirmed the interaction between CuO NPs and ranolazine. Figures 1a and 1b show the FTIR spectra of pure ranolazine and CuO NPs, respectively. Figure 1a shows many functional groups such as -NH stretching at 3327 cm ^-1^ and bending at 1589 cm ^-1^ ; the band at 1681 cm ^-1^ indicates the presence of -C=O in pure ranolazine while peaks at 2922 and 1465 cm ^-1^ are assigned to aliphatic (-C-H) stretching and bending, respectively. The FTIR spectrum of CuO NPs (Figure 1b) revealed similar vibrational bands, but the carbonyl group (-C=O) shown poor intensity. Furthermore, the band due to the -NH group is totally absent, which was present in Figure 1a, which confirmed that the -NH group is involved in the interaction between the drug and CuO NPs. The peak at 536 cm ^-1^ described the vibrations linked with CuO NPs [24], while Cu2 O peaks at 605 to 660 cm ^-1^ [25] were not observed in the present study, which clearly indicates the configuration of CuO NPs.

### 3.2. Scanning electron microscopy (SEM)

**Figure 2:**
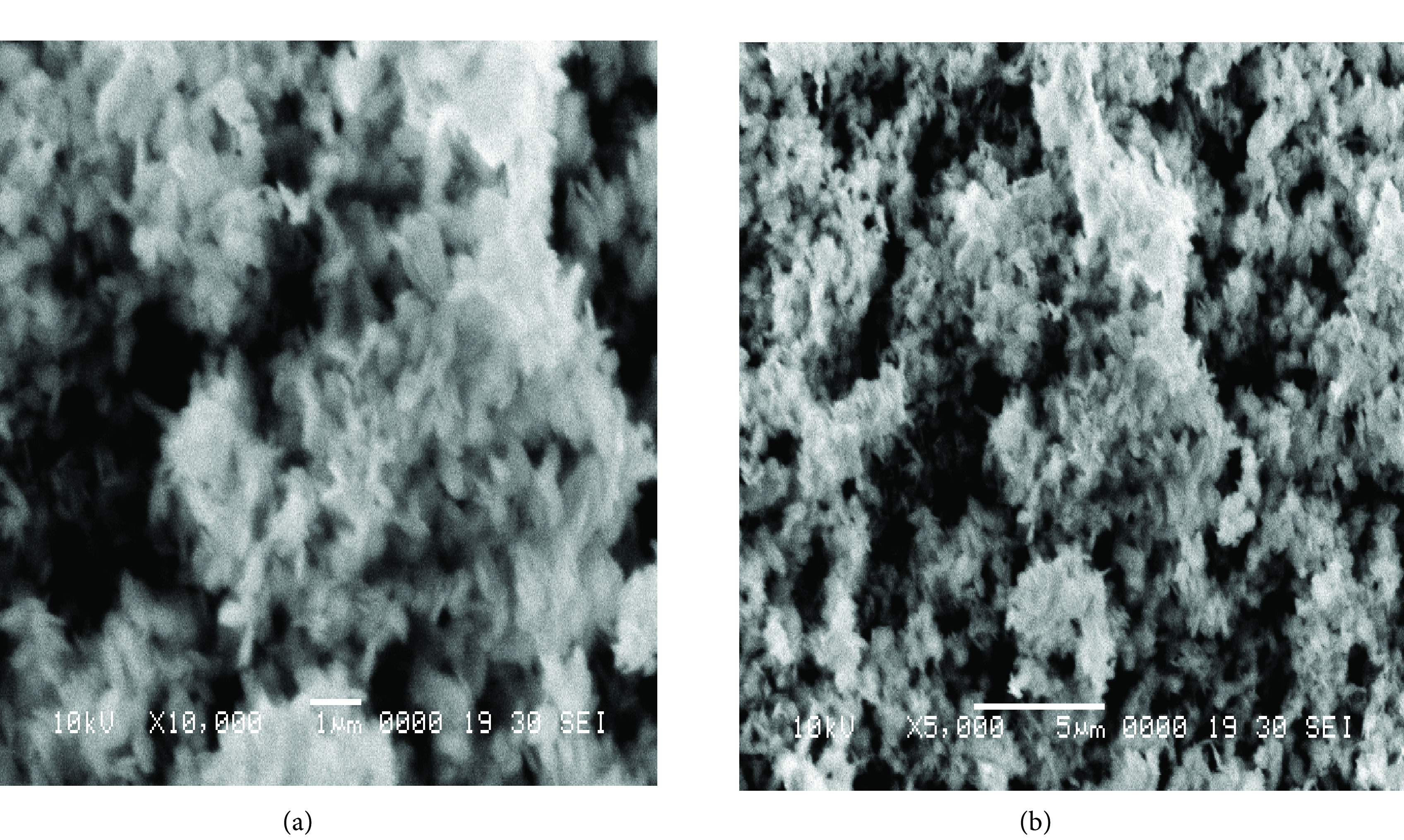
SEM images (a, b) of ranolazine-derived CuO NPs at low resolution (a) and high resolution (b), .

The surface characteristics of CuO NPs was studied by the help of SEM images as shown in Figures 2a and 2b at low resolution and high resolution, respectively, which demonstrated that the synthesized nanoparticles have rice-like structures.

### 3.3. X-ray diffractometry (XRD)

**Figure 3:**
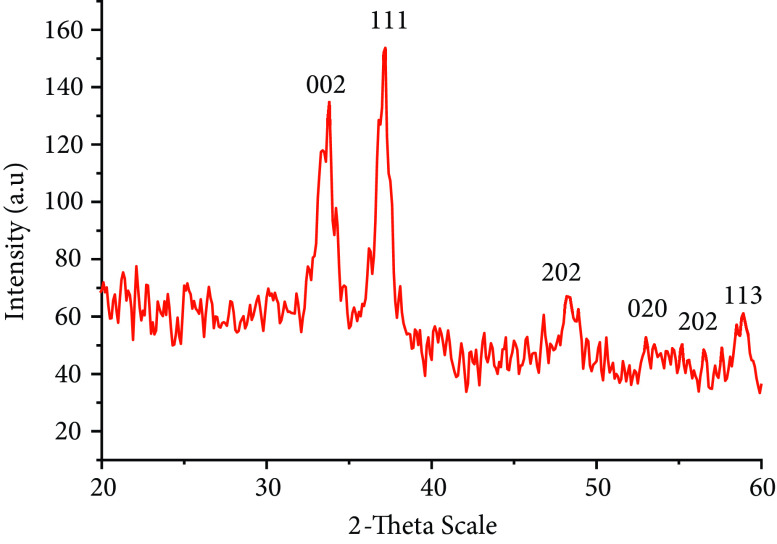
XRD pattern of functionalized CuO nanoparticles.

The crystalline structures and phase purities of ranolazine-functionalized CuO NPs were confirmed by XRD analysis. Figure 3 shows characteristic Miller indices (002), (111), (202), (020), (202), and (113) while lattice plane indices were observed at 2 theta values of 26.5◦ , 33.5◦ , 37.1◦ , 48.3◦ , 53.1◦ , 56.5◦ , and 59.1◦ , respectively. These CuO values demonstrated pure monoclinic crystalline structures and verified the JCPDS data (card no. 05-661). Additionally, Cu or Cu(OH) _2_ was not observed. The average size of CuO NPs was calculated to be about 12 nm and the particle range was from 9.3 to 18 nm by Scherrer’s equation:

D = (Kλ) /(β Cosθ) ,

where D is the average nanocrystallite size (nm) of nanoparticles, K is the Scherrer constant, λ is the X-ray wavelength (Cu Kα, 1.542 Å), β is the full-width at half-maximum intensity, and θ is half of the diffraction peak angle [26].

### 3.4. Catalytic activity of CuO NPs

**Figure 4:**
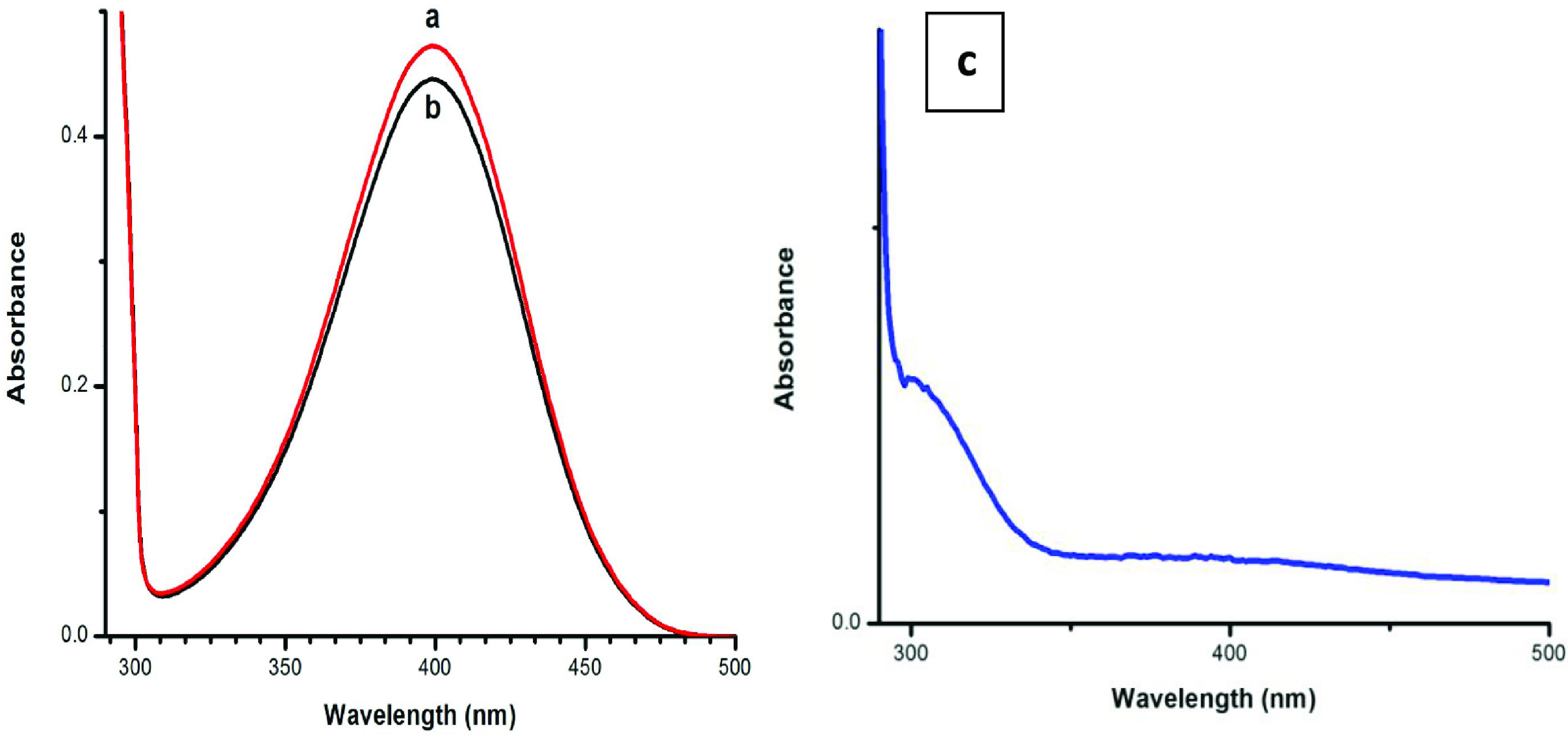
UV-Vis spectrum of pure 4-NP (a); slight reduction of 4-NP in the presence of only NaBH4 (b); complete reduction of 4-NP to 4-AP in the presence of NaBH4 and CuO NPs in both catalysis media at different time periods (c).

In the present work, we used CuO NPs as catalysts for the reduction of 4-NP in both homogeneous and heterogeneous media. To determine the catalytic response of CuO NPs in the presence of NaBH4 , Figure 4a represents the UV-Vis spectrum of pure 4-NP, Figure 4b shows slight reduction of 4-NP in the presence of only NaBH4 , and Figure 4c indicates complete reduction of 4-NP in the presence of NaBH4 and CuO NPs. The UV-Vis spectrometer monitored the reduction of 4-NP at regular intervals. In the absence of NPs, slight reduction of 4-NP was observed with NaBH4 , which is very clear in Figure 4b. We clearly see that after adding NaBH4 the solution color turned from light yellow to deep yellow due to the formation of the 4-nitrophenolate ions [27,28], while after addition of CuO NPs to the homogeneous medium, we observed 99% reduction of 4-NP to 4-AP in a few seconds as displayed in Figure 4c and almost complete reduction was observed for the heterogeneous medium but in a different time period. The proposed mechanism for catalytic conversion of 4-NP to 4-AP in the existence of NaBH4 is as follows:

**Figure:**
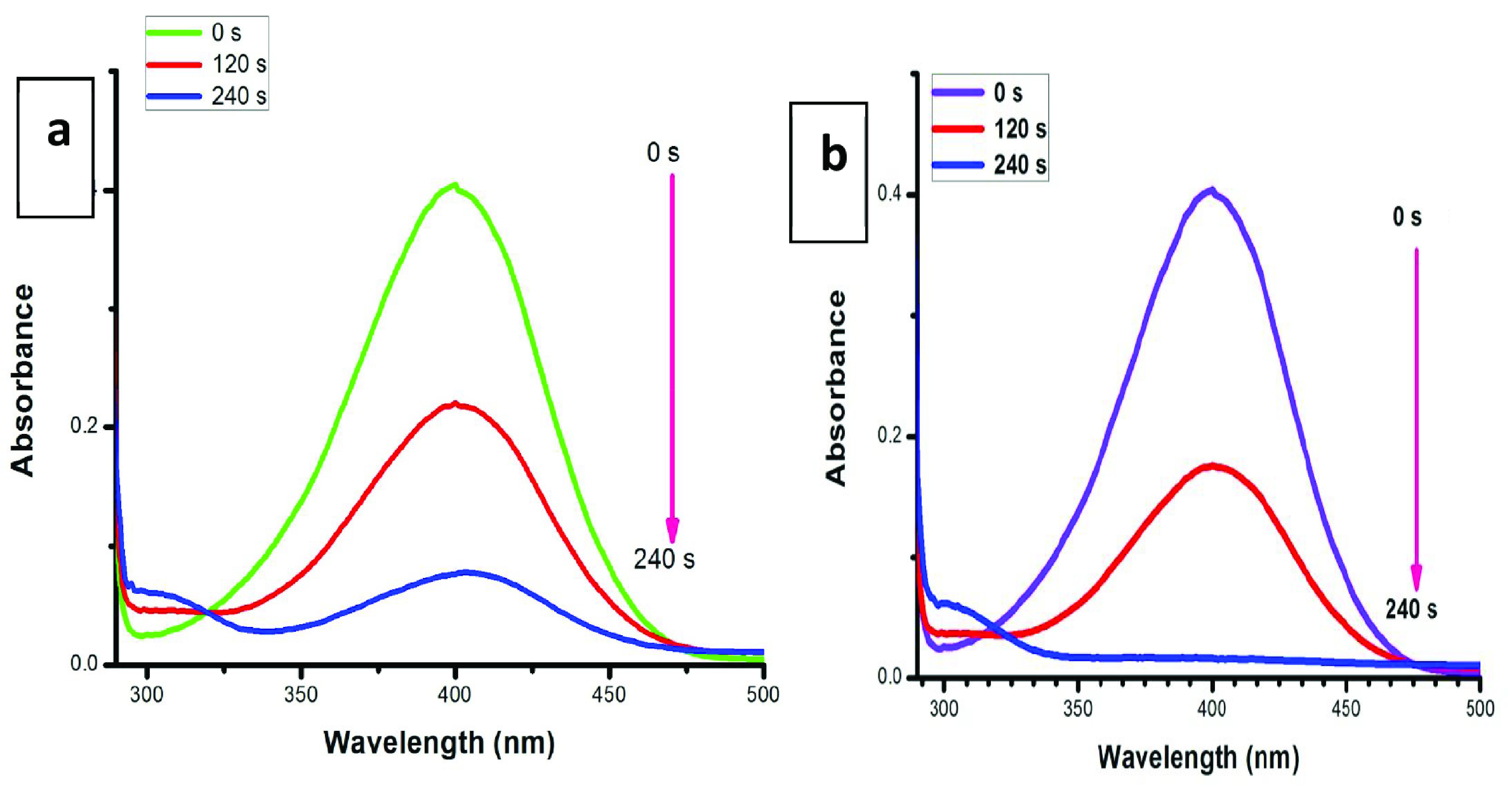


### 3.5. Homogeneous catalysis

**Figure 5:**
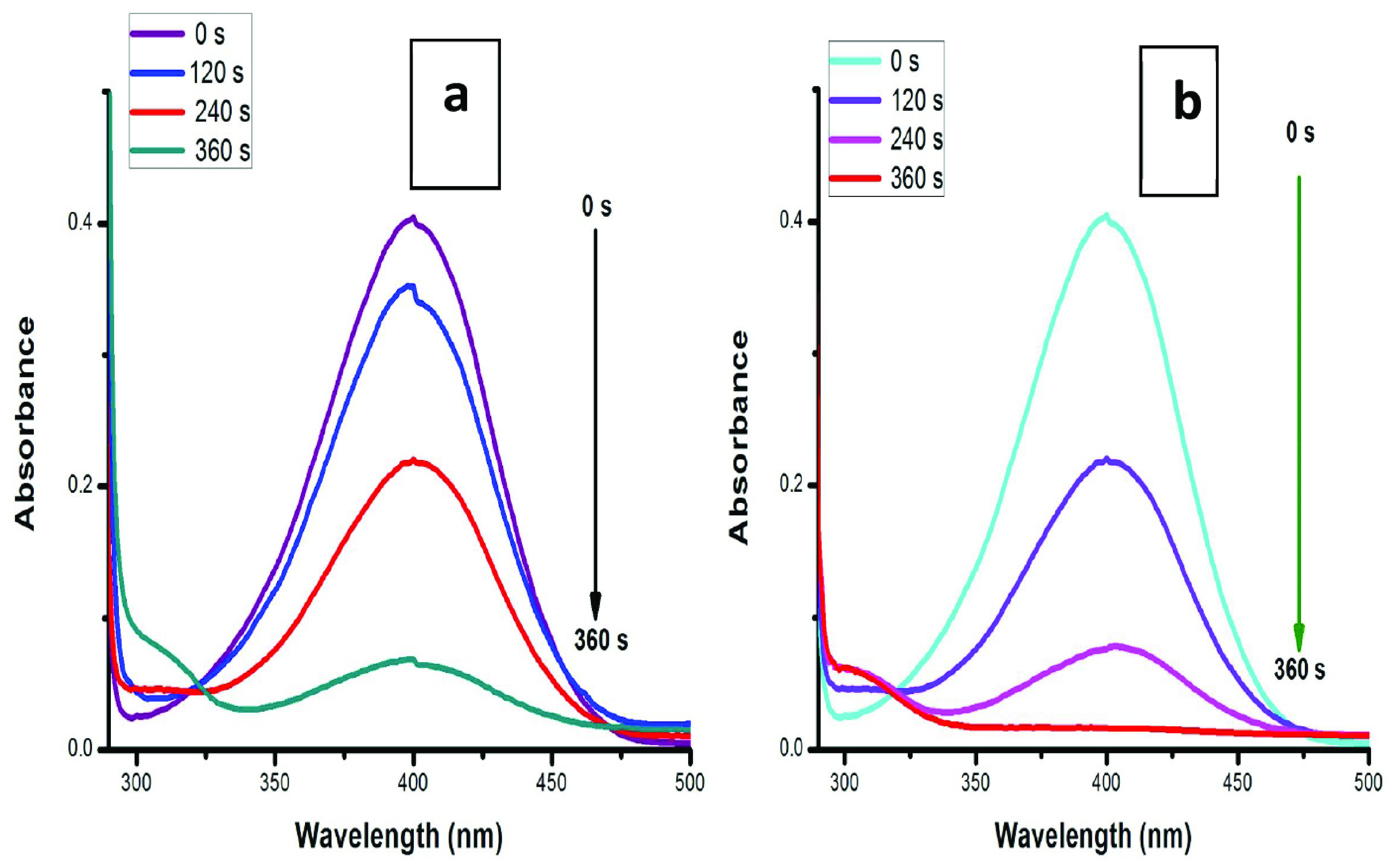
Homogeneous catalysis: effect of CuO NP dose on reduction of 4-nitrophenol (a) CuO NPs, 20 μL (0.1 mg mL ^-1^ ) ; (b) CuO NPs, 50 μL (0.25 mg mL ^-1^ ) .

Figure 5a indicates that when 20 μL (0.1 mg mL ^-1^ aqueous medium) of CuO NPs was added in the presence of NaBH4 , 64% reduction of 4-NP was experimentally observed in 240 s. However, when we increased the catalytic dose up to 50 μL (0.25 mg mL ^-1^ aqueous solution), we clearly observed 99% reduction of 4-NP in 240 s as shown in Figure 5b. As the concentration of CuO NPs increased, increased reduction of 4-NP was observed due to the presence of a more active surface area. On the other hand, we observed the appearance of a new peak at 305 nm, which confirmed the formation of 4-AP [29]. The clear effect of CuO NPs dose in the homogeneous medium is illustrated in Table 1.

**Table 1:** The effect of CuO NP amounts on 4-NP in homogeneous media.

CuO NP amount	Concentration of 4-NP	Reaction completion time	Catalytic reduction
20 μL (0.1 mg mL ^-1^ )	0.1 mM	240 s	64%
50 μL (0.25 mg mL ^-1^ )	0.1 mM	240 s	99%

### 3.6. Heterogeneous catalysis

**Figure 6:**
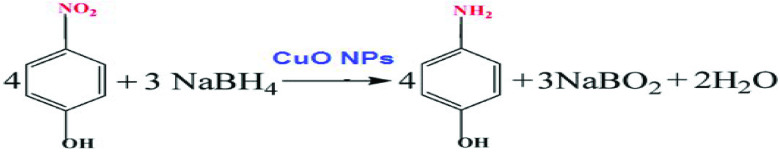
Heterogeneous catalysis: the effect of CuO NP dose on reduction of 4-NP: (a) CuO NPs at 0.2 mg, (b) CuO NPs at 0.5 mg.

In this study, preweighed glass strips with 0.2 mg of CuO NPs were used (Figure 6a) and the results showed 73% reduction of 4-NP in 360 s. When we used the other glass slides, assembled with 0.5 mg of CuO NPs, almost 99% reduction was observed for 4-NP within 360 s due to the presence of more CuO NPs and accessibility of more active sites (Figure 6b). On the other hand, the catalytic efficiency was not as effective as in the homogeneous phase due to blockage of various active surface sites due to their attachment to the glass slide. Therefore, a greater amount of CuO NPs was used as compared to homogeneous catalysis. The key benefit of heterogeneous catalysis is that we can reuse CuO NP catalysts for other cycles. The effect of CuO NP dose in the heterogeneous medium is illustrated in Table 2.

**Table 2:** The effect of CuO NP amounts on 4-NP in heterogeneous media.

CuO NP amount	Concentration of 4-NP	Reaction completion time	Catalytic reduction
0.2 mg	0.1 mM	360 s	73%
0.5 mg	0.1 mM	360 s	99%

### 3.7. Kinetic study

**Figure 7:**
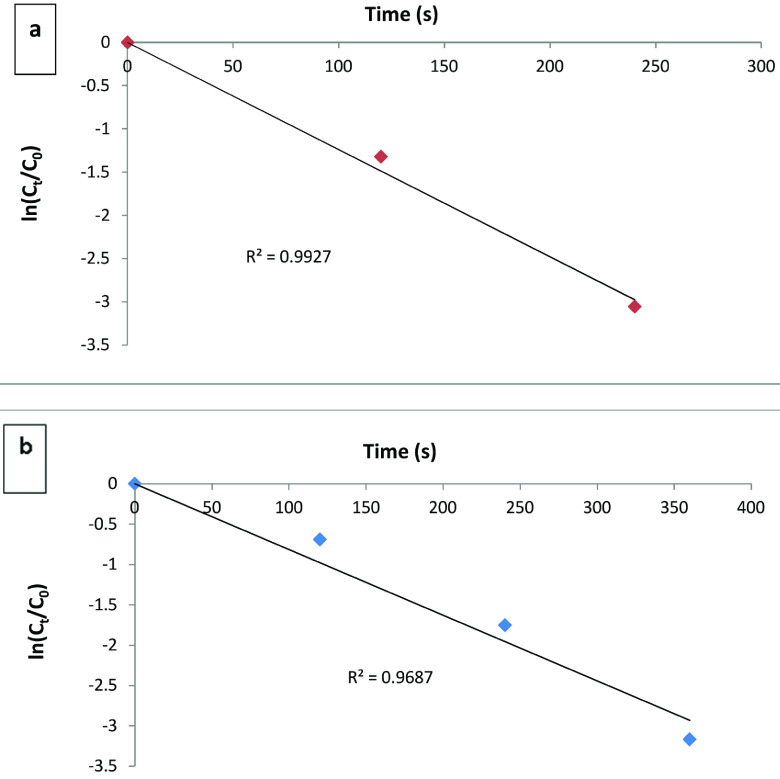
Kinetic plots (a) of ln(Ct /C0) vs. time (s) for homogenous catalysis for 0.25 mg mL ^-1^ and (b) ln(Ct /C0) vs. time (s) for heterogeneous catalysis for 0.5 mg of CuO NPs.

The reduction reaction of 4-NP can be carried out with NaBH4 , but it is remarkable to observe that the rate of reaction is independent of the amount of NaBH4 . The kinetic study was confirmed by plotting ln(Ct /C0) versus reduction time (s), which follows a pseudo-first-order reaction [8]. Figure 7a shows the homogeneous catalysis plot between the ln(Ct /C0) of 4-NP and time (s) at 0.25 mg mL ^-1^ (50 μL) of CuO NPs. Reaction rate was calculated from the slope of the excellent linearly fitted plot and found to be 1.3 × 10 ^-2^ s ^-1^ for homogeneous catalysis (Figure 7a). The rate of reaction at 0.5 mg of CuO NPs was found to be 8.8 × 10 ^-3^ s ^-1^ for heterogeneous medium as in Figure 7b. These results indicated that with increased CuO NP dose, the rate of reaction was increased and almost complete catalytic reduction of 4-NP occurred in less than 400 s.

### 3.8. Catalytic reusability

**Figure 8:**
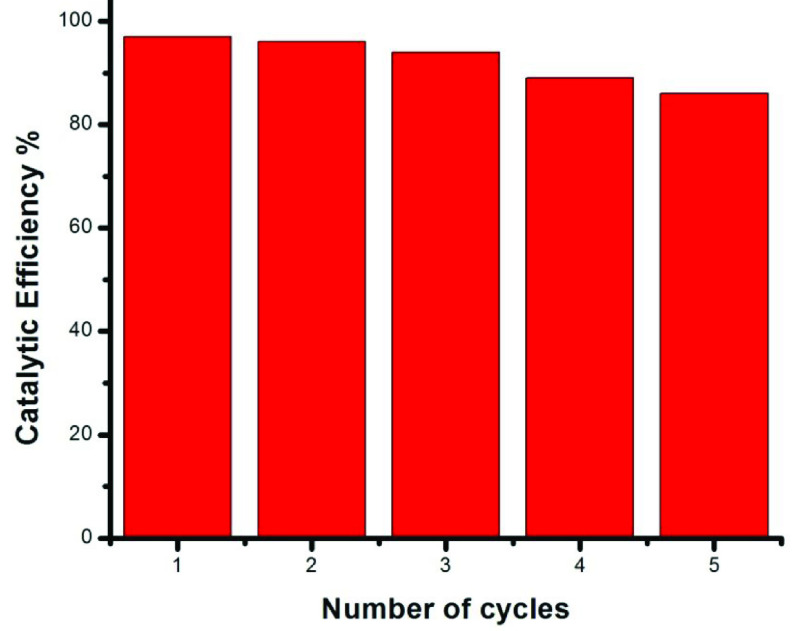
Catalytic efficiency of CuO NPs in five cycles.

The reusability of CuO NPs in homogeneous medium is not possible. However, in the case of the heterogeneous medium, 5 cycles were effectively observed with negligible loss of catalytic efficiency as shown in Figure 8. CuO NPs have excellent catalytic efficiency and are therefore suitable for economical purposes.

### 3.9. Comparative studies

The smaller size of NPs exhibits better catalytic efficiency because of the higher surface area. Compared to gold, silver, and palladium NPs, the cost for preparation of Cu NPs is obviously low. Majumdar et al. reported the preparation of Au NPs from bark extract of Mimusops elengi. HRTEM was used to determine average particle size of about 11 nm, but due to limited stability of the suspensions these NPs could not be reused [30]. Aitenneite et al. prepared Ag NPs using Phoenix dactylifera L. leaf extract and the average particle size was calculated as approximately 40 nm. Perhaps due to greater particle size, the performance of these NPs was poor and they required 18 min for reduction of 4-NP [31]. Other authors reported Silibum marianum as a capping agent for the preparation of Pd NPs and the average size was found to be about 20 nm. The complete catalytic reduction of 4-NP to 4-AP was observed in 17 min [32]. On the other hand, the permanence of metallic NPs is a serious issue and also causes catastrophic aggregation and deactivation of catalytic performance. Saikia et al. reported composites of Cu with Pd NPs for conversion of 4-NP to 4-AP, but due to complexity and limited stability of suspensions, these NPs would be limited for use [33]. Another work reported the coupling of Ag with TiO _2_ by sol-gel method but utilized a higher volume of surfactant during synthesis [34]. Castañeda et al. prepared TiO _2_ by sol-gel method and catalytic reduction of 4-NP to 4-AP was completed in 240 min [35,36]. In the current study, the simplest and conventional hydrothermal protocol for preparing CuO NPs was carried out using a cheaper biodegradable drug (ranolazine) as a capping agent. Results of the present study indicated that the environmentally friendly, economic, rapid, and simple protocol for the synthesis of CuO NPs was performed for the catalytic reduction of 4-NP into 4-AP. Table 3 provides the comparison of the current study with the already reported works on the catalytic reduction of 4-NP to 4-AP by different metals, metal oxides, and composite NPs.

**Table 3:** The comparative results on the reduction of 4-NP by metal nanoparticles.

Catalyst	Size of NPs (nm)	Conc. of 4-NP (mM)	Reaction time (s)	Catalytic rate constant (s ^-1^ )	Type of catalysis	Remarks	Ref.
Au-NPs	11	0.05	480	6.5 × 10 ^-3^	Homogeneous	Expensive, Ênot reusable	[25]
Ag NPs	~40	1	1080	2.4 × 10 ^-3^	Homogeneous	Expensive, Ênot reusable	[26]
Pd NPs	< 20	20	1020	Not calculated	Homogeneous	Expensive NPs, not reusable	[27]
Cu@Pd NPs	3	0.1	720	5.8 × 10 ^-3^	Heterogeneous	Complicated, Êslow reduction	[28]
Ag/TiO _2_ NPs	~11	0.004	210	1.9 × 10 ^-2^	Heterogeneous	Complicated NPs	[29]
TiO _2_	10	0.0001	14400	2.2 × 10 ^-2^	Heterogeneous	Very slow, not economical	[30]
CuO NPs	12	0.1	240	1.3 × 10 ^-2^	Homogeneous	Simplest, cheapest, rapid	Present work
			360	8.8 × 10 ^-3^	Heterogeneous		

### 3.10. Conclusions

A simple and convenient hydrothermal protocol was used for the synthesis of ranolazine-derived CuO NPs. Various characterization techniques such as UV-Vis, SEM, XRD, and FTIR spectroscopy were employed to study the surface topography and catalytic behavior of CuO NPs. The straightforward, rapid, and economic approach was applied for the synthesis of smaller sizes of CuO NPs, which provided more active sites for catalytic reduction of 4-NP to 4-AP. The catalytic process for heterogeneous catalysis is not more effective as in the case of homogeneous catalysis because several active sites of CuO NPs were blocked due to their attachment to glass strips. However, the advantage of heterogeneous catalysis is that CuO NPs can be reused as catalysts for several cycles. These CuO NPs are highly economical and nontoxic, with excellent efficiency for catalytic reduction of 4-NP in both homogeneous and heterogeneous media.
